# Diagnostic and Therapeutic Challenges in Herpes Simplex Virus-Triggered Anti-N-Methyl-D-Aspartate Receptor Encephalitis Presenting as Acute Psychiatric Illness in a Young Woman: A Case Report

**DOI:** 10.7759/cureus.110962

**Published:** 2026-06-16

**Authors:** Pratik Rathi, Fahad Idrees Shaikh, Tanuja Manohar, Arvind Agrawal, Nishka Tiwari

**Affiliations:** 1 General Medicine, Lata Mangeshkar Hospital, N.K.P. Salve Institute of Medical Sciences and Research Centre, Nagpur, IND; 2 Medical Education, Lata Mangeshkar Hospital, N.K.P. Salve Institute of Medical Sciences and Research Centre, Nagpur, IND; 3 Medicine, Lata Mangeshkar Hospital, N.K.P. Salve Institute of Medical Sciences and Research Centre, Nagpur, IND; 4 Internal Medicine, Lata Mangeshkar Hospital, N.K.P. Salve Institute of Medical Sciences and Research Centre, Nagpur, IND

**Keywords:** anti-nmdar encephalitis, autoimmune encephalitis, hsv-1 pcr, hsv encephalitis, overlap syndrome, psychiatric manifestations

## Abstract

Anti-N-methyl-D-aspartate receptor (anti-NMDAR) encephalitis is a severe form of autoimmune encephalitis. Symptoms include psychiatric manifestations, seizures, dyskinesias, autonomic instability, and altered consciousness. Recently, Herpes simplex virus (HSV) encephalitis has been recognised as a major cause of secondary autoimmune encephalitis. We report the case of a 23-year-old woman who developed behavioural changes and altered sensorium. She was initially suspected of having a primary psychiatric disorder. Subsequent tests showed the presence of anti-NMDAR antibodies in the cerebrospinal fluid (CSF), and positivity for HSV-1 was confirmed by real-time polymerase chain reaction (PCR). Despite receiving high-dose corticosteroids, IVIG, plasmapheresis, rituximab, acyclovir, and antiviral therapy, the patient was admitted to the ICU, where she stayed for a prolonged period due to respiratory failure, septic shock, and acute kidney injury. This case highlights the diagnostic challenge between viral and autoimmune encephalitis and underlines that patients with acute neuropsychiatric symptoms require early recognition, repeated CSF testing, and multidisciplinary management.

## Introduction

Anti-N-methyl-D-aspartate receptor (anti-NMDAR) encephalitis is an antibody-mediated autoimmune encephalitis caused by immunoglobulin G (IgG) antibodies directed against the GluN1 (NR1) subunit of the NMDA receptor, resulting in receptor internalisation and synaptic dysfunction [1]. It is now recognised as one of the most common forms of autoimmune encephalitis and predominantly affects children and young adults, particularly females [2]. Clinically, anti-NMDAR encephalitis follows a characteristic course beginning with prominent psychiatric manifestations, including behavioural changes, agitation, psychosis, catatonia, anxiety, and cognitive dysfunction, followed by seizures, movement disorders, autonomic instability, impaired consciousness, and respiratory failure [3]. Early diagnosis and timely initiation of immunotherapy have been associated with improved neurological outcomes [3]. Among all subtypes of Herpes simplex virus (HSV) encephalitis, HSV-1 is the most common subtype and is increasingly recognised as a major trigger for secondary autoimmune encephalitis [4]. HSV-mediated neuronal destruction in herpes simplex encephalitis is believed to expose synaptic antigens, leading to the production of autoimmune antibodies against NMDA receptors and the subsequent development of secondary autoimmune encephalitis [4]. Immunotherapeutic strategies such as corticosteroids, intravenous immunoglobulin, plasma exchange, and second-line agents such as rituximab play an important role in the management of autoimmune encephalitis, particularly in severe or refractory cases [5].

According to previous studies, anti-NMDAR antibodies may appear days to weeks after the initial episode of HSV encephalitis, supporting the concept of HSV-triggered brain autoimmunity [6]. Relapses and severe disease courses requiring escalation of therapy have also been reported, underscoring the importance of close follow-up and continued clinical vigilance [7]. Antibody-mediated encephalitis constitutes a heterogeneous group of disorders characterised by overlapping infectious and autoimmune mechanisms, creating significant diagnostic and therapeutic challenges [8]. Because the initial manifestations are often predominantly psychiatric, affected patients may initially be misdiagnosed with a primary psychiatric disorder, leading to delays in diagnosis and treatment [8]. HSV-triggered anti-NMDAR encephalitis represents a clinically important overlap syndrome requiring simultaneous antiviral therapy and aggressive immunomodulatory treatment [8]. We present a diagnostically challenging case of HSV-triggered anti-NMDAR encephalitis in a young woman who initially presented with acute psychiatric manifestations but was subsequently found to have concurrent HSV-1 polymerase chain reaction (PCR) positivity and cerebrospinal fluid (CSF) anti-NMDAR antibodies. Despite aggressive antiviral and multimodal immunotherapy, the patient developed severe refractory neurological disease requiring prolonged intensive care support, highlighting the diagnostic delay and therapeutic challenges associated with this overlap syndrome.

## Case presentation

A 23-year-old previously healthy female initially presented with a diffuse headache, which was insidious in onset, progressively worsening, and associated with sleep disturbances, photophobia, and recurrent vomiting (Day 1). Initially, the patient received symptomatic treatment at a peripheral healthcare centre. During the subsequent two days, progressive behavioural abnormalities, including irritability, emotional lability, social withdrawal, self-muttering, agitation, and insomnia, were observed by the patient’s relatives. Over the next few days, her condition deteriorated with increasing confusion and altered sensorium. By Day 3, she had become disoriented and exhibited fluctuating attention, impaired recent memory, and reduced verbal interaction. By Day 5, she was transferred to another institution where a primary psychiatric illness was suspected, and psychiatric treatment was initiated. However, despite psychiatric management, the patient's mental status progressively worsened, raising suspicion for another underlying cause. By Day 8, she developed fever, neck stiffness, and worsening consciousness. Persistent fever with chills and neck stiffness suggested the need for further evaluation for an infectious cause, and she was transferred to our institution. On admission, the patient was febrile (101°F), anxious, disoriented, and unable to follow verbal commands. Vital signs revealed tachycardia (130/min), blood pressure of 160/80 mmHg, respiratory rate of 24/min, and oxygen saturation of 95% on room air. Mental status examination revealed marked impairment in higher cortical functions. She was disoriented to time, place, and person and demonstrated impaired attention and recent memory. Speech became progressively sparse and incoherent, eventually progressing to mutism during periods of catatonia. Behavioural abnormalities included agitation, emotional instability, self-muttering, and poor interaction with family members. Catatonic features fluctuated in severity and consisted of mutism, staring episodes, reduced spontaneous movements, posturing, negativism, and poor responsiveness to verbal commands. As the disease progressed, abnormal involuntary movements became increasingly prominent. Characteristic orofacial dyskinesias consisting of repetitive lip-smacking, tongue protrusion, chewing movements, and facial grimacing were noted. Intermittent limb and truncal dyskinetic movements were also observed. Episodes of autonomic dysfunction occurred during the severe phase of illness and were characterised by persistent fever spikes, tachycardia, fluctuating blood pressure, temperature instability, and variable heart rate, findings consistent with severe anti-NMDA receptor encephalitis.

Neurological examination revealed an altered sensorium with poor cooperation. Neck stiffness and a positive Kernig's sign were present, indicating meningeal irritation. Cranial nerve examination demonstrated equal and reactive pupils, preserved extraocular movements, and no facial asymmetry. Muscle bulk was preserved, and no focal cranial nerve deficit was identified. Motor examination during the early phase was limited because of poor cooperation, although no obvious focal weakness was appreciated. Deep tendon reflexes were preserved, plantar responses were flexor bilaterally, and sensory examination was grossly unremarkable but difficult to assess because of the patient's encephalopathic state. The presence of fever, neck stiffness, a positive Kernig's sign, and altered sensorium indicated an encephalitic process rather than a primary psychiatric disorder and prompted further cerebrospinal fluid analysis and neuroimaging. On Day 9, routine haematological investigations were performed, as shown in Table 1. Cerebrospinal fluid analysis demonstrated normal glucose (76 mg/dL) and protein (25 mg/dL) concentrations with acellular fluid. 

**Table 1:** Significant laboratory trends during hospitalization.

Parameter	Significant Observation	Biological Reference Interval	Clinical Significance	Therapeutic Implication
Total leukocyte count	43,130 cells/mm³	4,000–11,000 cells/mm³	Inflammation/sepsis	Antibiotic escalation
Serum creatinine	2.63 mg/dL	0.6–1.1 mg/dL	Acyclovir nephrotoxicity suspected	Forced alkaline diuresis
Procalcitonin	5 ng/mL	<0.1 ng/mL	Septic episodes	ICU monitoring
Arterial blood gas	Episodes of respiratory alkalosis	pH 7.35–7.45; PaCO₂ 35–45 mmHg	Respiratory instability	Ventilatory support
Urine routine examination	Microscopic hematuria present	Absent (0–2 RBCs/HPF)	Suggestive of crystal nephropathy	Renal monitoring

Although these findings were not suggestive of active central nervous system inflammation, anti-NMDAR antibodies in the cerebrospinal fluid were positive by immunofluorescence assay, supporting the diagnosis of autoimmune encephalitis, as shown in Table 2. 

**Table 2:** Important diagnostic findings and clinical significance.

Investigation	Findings	Biological Reference Interval
MRI brain	No significant abnormalities	No focal or diffuse abnormalities detected
CSF glucose	76 mg/dL	45–80 mg/dL
CSF cell count	1 cell/mm³	0–5 cells/mm³

Magnetic resonance imaging of the brain was performed, which revealed no significant structural abnormalities (Table 2). Despite these apparently reassuring findings, the persistence of psychiatric manifestations, altered sensorium, dyskinesias, seizures, and autonomic instability maintained a high clinical suspicion for autoimmune or infectious encephalitis. Therefore, normal neuroimaging and an acellular cerebrospinal fluid were not considered sufficient to exclude encephalitis.

**Figure 1:**
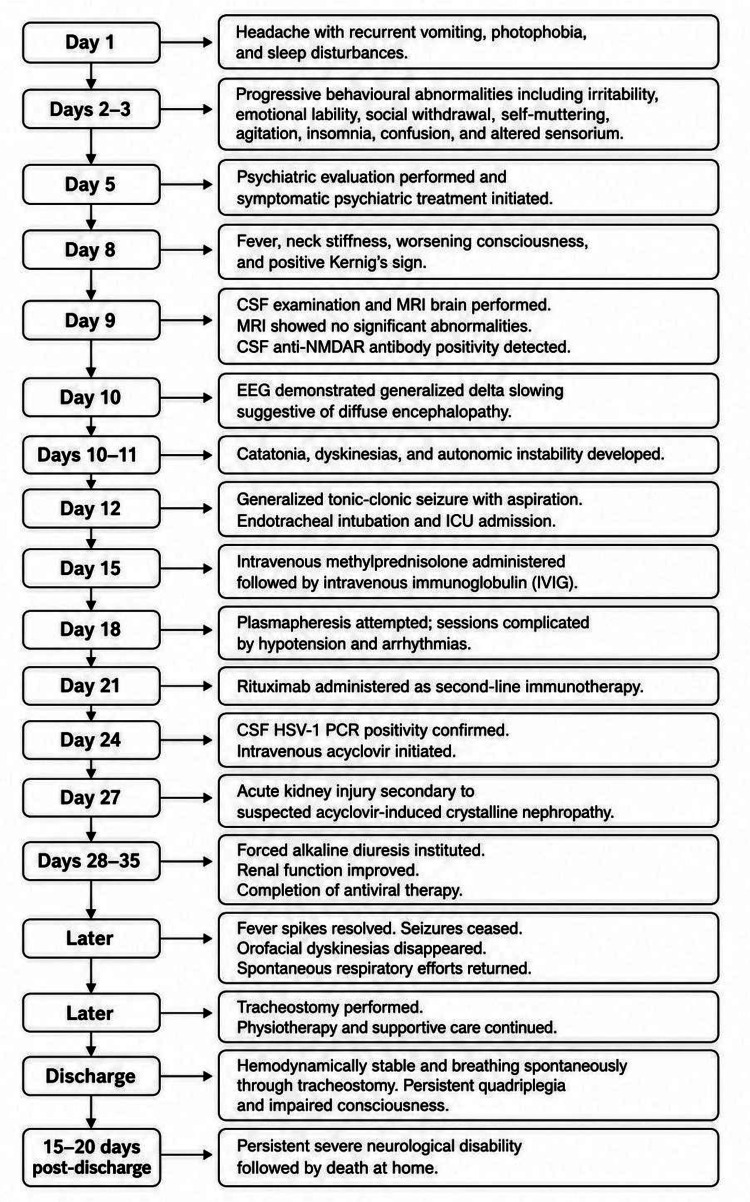
Clinical timeline demonstrating symptom progression, diagnostic, investigations, therapeutic interventions, complications, and outcomes in HSV-triggered anti-NMDAR encephalitis. CSF: cerebrospinal fluid, MRI: magnetic resonance imaging, anti-NMDAR: anti-N-methyl-D-aspartate receptor, EEG: electroencephalogram, ICU: intensive care unit, IVIG: intravenous immunoglobulin, HSV-1: herpes simplex virus type 1, PCR: polymerase chain reaction.

The clinical timeline demonstrating symptom progression, diagnostic investigations, therapeutic interventions, complications, and outcomes in HSV-triggered anti-NMDAR encephalitis is shown in Figure 1. 

**Figure 2:**
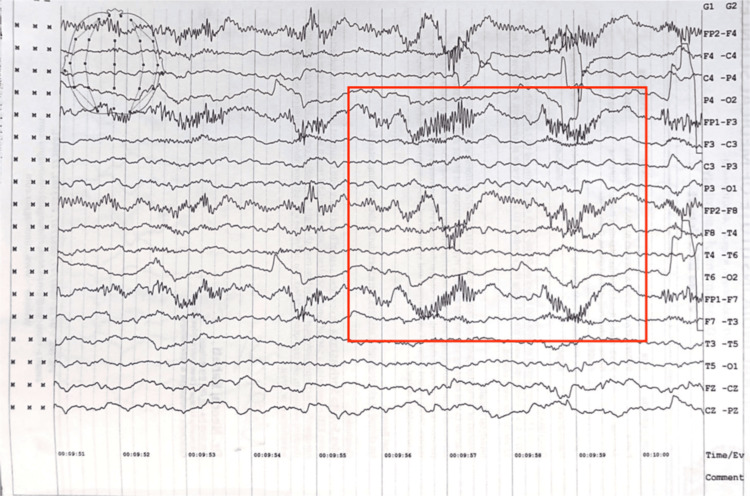
Electroencephalography (EEG) showing generalized delta slowing which was suggestive of severe diffuse encephalopathic dysfunction without epileptiform discharges in autoimmune encephalitis.

Electroencephalography subsequently demonstrated generalised delta slowing consistent with diffuse encephalopathic dysfunction. Despite normal MRI, the abnormal EEG findings strengthened the suspicion of autoimmune encephalitis and represented an important diagnostic clue (Figure 2). 

Further CSF studies demonstrated positivity for anti-NMDA receptor antibodies by immunofluorescence assay, as shown in Table 3. On Day 12, she experienced generalised tonic-clonic seizures, which were complicated by aspiration, necessitating endotracheal intubation, and she was then shifted to the intensive care unit. Immunotherapy was initiated empirically after suspicion of autoimmune encephalitis. On Day 15, intravenous corticosteroids were administered, followed by intravenous immunoglobulin therapy. Despite treatment, encephalopathy, dyskinesias, and catatonic features persisted. Consequently, given the refractory nature of the disease, plasmapheresis was performed on Day 18 under nephrology supervision. However, the initial two plasmapheresis sessions were complicated by hypotension and arrhythmias requiring noradrenaline support, ultimately leading to discontinuation of further exchange therapy. Rituximab infusion was subsequently administered as second-line immunotherapy on Day 21. Although seizure frequency gradually decreased following treatment escalation, significant neurological improvement was not achieved, and the patient continued to exhibit severe encephalopathy. On Day 24, CSF PCR showed HSV-1 positivity, thereby establishing the diagnosis of HSV-triggered anti-NMDAR overlap syndrome, and intravenous acyclovir therapy was started along with the ongoing immunomodulatory treatment (Table 4). 

**Table 3:** CSF NMDA receptor antibody report: immunofluorescence assay illustrating CSF positivity for NMDA receptor antibodies. CSF: cerebrospinal fluid, NMDA: N-methyl-D-aspartate.

Investigation	Observed Value	Biological Reference Interval	Method
NMDA receptor antibody, CSF (CSF immunofluorescence assay (IFA))	Positive	Negative	Immunofluorescence assay (IFA)

**Table 4:** HSV-1 PCR report: real-time PCR assay confirming detection of HSV-1 in cerebrospinal fluid. HSV-1: herpes simplex virus type 1, PCR: polymerase chain reaction.

Test	Result	Interpretation
Herpes simplex virus 1	Detected	Target DNA detected
Herpes simplex virus 2	Not detected	Target DNA not detected

During treatment, the patient developed acute kidney injury on Day 27, which was suspected to be secondary to acyclovir-induced crystalline nephropathy, as evidenced by rising serum creatinine levels and microscopic haematuria (Table 4). Forced alkaline diuresis and supportive measures were then instituted, following which renal function gradually improved. The planned course of antiviral therapy was completed successfully. Malignancy screening was also performed, including ultrasonography of the abdomen and pelvis and measurement of serum CA-125 levels, which did not reveal evidence of ovarian teratoma or an underlying neoplasm (Table 5). 

**Table 5:** Malignancy screening confirmed no evidence of ovarian teratoma or underlying neoplasm. USG: ultrasonography.

Test	Result	Reference Range
CA-125	25 U/mL	<35 U/mL
USG abdomen/pelvis	No ovarian teratoma detected	No ovarian or pelvic mass detected

With treatment, fever spikes subsided, seizures completely ceased, and the prominent orofacial dyskinesias gradually disappeared. Spontaneous respiratory efforts returned, and ventilatory support was successfully weaned. However, despite resolution of seizures and improvement of systemic complications, no substantial neurological recovery was observed. The patient exhibited spontaneous eye movements but remained unable to follow commands or interact purposefully with her surroundings. Because of prolonged ventilatory dependence, a tracheostomy was then performed, and supportive care, including tracheostomy care, physiotherapy, nutritional support, and rehabilitation, was continued. At the time of discharge, the patient was haemodynamically stable and breathing spontaneously through the tracheostomy. However, severe neurological deficits persisted, with quadriplegia and impaired consciousness. Despite supportive management, the patient died approximately 15-20 days after discharge from the hospital. 

**Table 6:** Differential Diagnosis Considered during hospital course. HSV: herpes simplex virus, anti-NMDAR: anti-N-methyl-D-aspartate receptor, CSF: cerebrospinal fluid, PCR: polymerase chain reaction.

Differential Diagnosis	Supporting Features	Features Against Diagnosis
Primary psychiatric disorder	Behavioural disturbances and catatonic features	Presence of fever, meningism, seizures
HSV encephalitis	Fever and altered sensorium	Concurrent anti-NMDAR positivity
Autoimmune encephalitis	Dyskinesias and psychiatric manifestations	HSV positivity suggested overlap syndrome
Tubercular meningitis	Subacute presentation	CSF findings not supportive
HSV-triggered anti-NMDAR encephalitis	Positive anti-NMDAR antibody and HSV-1 PCR	None

## Discussion

Anti-NMDAR encephalitis is characterized by prominent psychiatric manifestations, seizures, movement disorders, autonomic instability, and altered consciousness. These psychiatric manifestations are usually the first signs of anti-NMDAR encephalitis and may mimic primary psychiatric illness, which can cause delays in appropriate neurological evaluation [9]. Similarly, in the present case, the patient was initially managed as a psychiatric case because of her behavioural changes and catatonic features until the subsequent development of fever, altered sensorium, seizures, and dyskinesias, which indicated an underlying organic neurological disorder. An important feature of this case was the discrepancy between neuroimaging and electrophysiological findings [10]. Despite severe neurological dysfunction, MRI of the brain remained normal, whereas EEG demonstrated generalised delta slowing consistent with diffuse encephalopathy. Studies have shown that MRI findings may remain unremarkable in anti-NMDAR encephalitis despite profound clinical manifestations [10]. Therefore, clinicians should not be falsely reassured by normal neuroimaging when most of the characteristic clinical features are present. In our patient, the combination of normal MRI findings and abnormal EEG findings represented an important diagnostic clue supporting autoimmune encephalitis. Another notable aspect was the coexistence of HSV-1 and anti-NMDAR antibody positivity, which supported the diagnosis of HSV-triggered anti-NMDAR encephalitis. Previous studies have shown that HSV encephalitis is an important trigger for secondary autoimmune encephalitis and have generally reported a biphasic course with delayed appearance of anti-NMDAR antibodies [11]. However, in the present case, anti-NMDAR antibody positivity preceded confirmation of HSV-1 PCR positivity, suggesting that infectious and autoimmune mechanisms may coexist during the same episode. This observation highlights the importance of maintaining clinical suspicion even in the absence of the classical biphasic pattern [11].

Furthermore, despite initially acellular cerebrospinal fluid and normal MRI findings, generalised slowing on EEG and the presence of anti-NMDAR antibodies supported autoimmune encephalitis. Current recommendations emphasize that EEG abnormalities may provide supportive diagnostic evidence when structural imaging is unrevealing and that repeat cerebrospinal fluid evaluation should be considered when clinical suspicion persists [12]. Additional parameters such as anti-NMDAR antibody titres, IgG index, and oligoclonal bands were unavailable in our patient. In patients with persistent clinical suspicion despite initially non-specific findings, repeat cerebrospinal fluid analysis for viral PCR and neuronal antibodies should be considered according to the evolving clinical course. Recognition of such overlap syndromes is clinically important because management frequently requires treatment directed against both the infectious trigger and the autoimmune process. In our patient, antiviral therapy alone would likely have been insufficient, necessitating escalation from corticosteroids and intravenous immunoglobulin to plasmapheresis and rituximab, in addition to intravenous acyclovir. This observation highlights the importance of combined antiviral and immunomodulatory therapy in patients with HSV-triggered anti-NMDAR encephalitis [12].

Despite aggressive multimodal therapy, the patient experienced various complications, including prolonged ventilatory dependence, septic shock, and acute kidney injury suggestive of acyclovir-associated crystalline nephropathy. These findings emphasise the importance of regular renal function monitoring during acyclovir administration and the need for multidisciplinary supportive care, including early airway planning, tracheostomy, physiotherapy, and rehabilitation. Malignancy screening was negative in this patient. Since ovarian teratoma is strongly associated with anti-NMDAR encephalitis in young females, exclusion of an underlying neoplasm remains an essential component of evaluation [13]. In summary, several converging findings supported the diagnosis of HSV-triggered anti-NMDAR encephalitis. These included an initial psychiatric presentation, progression to encephalopathy and seizures, characteristic dyskinesias and autonomic instability, generalised EEG slowing despite normal MRI findings, anti-NMDAR antibody positivity, subsequent HSV-1 PCR positivity, and the absence of ovarian teratoma. The need for combined antiviral therapy, multimodal immunotherapy, prolonged intensive care support, and management of treatment-related complications further underscored the complexity of this overlap syndrome. Overall, this case underscores the importance of considering autoimmune encephalitis in young patients who present with sudden psychiatric manifestations, particularly when these symptoms are accompanied by seizures, dyskinesias, autonomic instability, or altered consciousness.

## Conclusions

This case highlights the diagnostic and therapeutic complexity of HSV-triggered anti-NMDAR encephalitis and demonstrates that infectious and autoimmune mechanisms may coexist during a single severe episode. In our patient, the coexistence of anti-NMDAR antibody positivity and subsequent HSV-1 PCR positivity, together with generalised EEG slowing despite normal MRI findings and initially acellular cerebrospinal fluid, represented important diagnostic clues supporting the diagnosis of an overlap syndrome. These findings emphasise that clinicians should maintain suspicion for autoimmune encephalitis even when routine neuroimaging and basic cerebrospinal fluid parameters are not strikingly abnormal. This case also underscores the need to balance aggressive treatment of both infectious and autoimmune components with vigilant monitoring for treatment-related complications and prolonged multidisciplinary supportive care. It further highlights the importance of integrating clinical findings with electroencephalographic, virological, and immunological investigations, particularly in patients presenting with acute psychiatric manifestations accompanied by seizures, dyskinesias, autonomic instability, or altered consciousness. Although the temporal association strongly suggests HSV infection as a trigger for secondary autoimmune encephalitis, a causal relationship cannot be definitively established from a single case report. Furthermore, the absence of certain ancillary cerebrospinal fluid parameters and limited long-term follow-up represent important limitations. Nevertheless, this case contributes to the growing evidence regarding HSV-triggered anti-NMDAR encephalitis and reinforces the importance of early recognition, combined antiviral and immunomodulatory therapy, and careful monitoring for complications in these challenging overlap syndromes.
